# Case report: A healthy baby achieved after preimplantation genetic testing from an infertile woman with hereditary leiomyomatosis and renal cell cancer syndrome

**DOI:** 10.3389/fmed.2024.1400694

**Published:** 2024-06-12

**Authors:** Qianhui Hu, Qing Zhang, Mengxi Guo, Haixia Ding, Ji Xi, Meiling Zhang, Min Wang, Lin Zhang, Shuyuan Li, Dandan Wu, Wen Li

**Affiliations:** The International Peace Maternity and Child Health Hospital, School of Medicine, Shanghai Jiao Tong University, Shanghai, China

**Keywords:** case report, hereditary leiomyomatosis and renal cell cancer, PGT-M, embryo transplantation, uterine leiomyomas

## Abstract

**Background:**

Hereditary leiomyomatosis and renal cell cancer (HLRCC) is a rare autosomal dominant inheritable disease caused by Fumarate hydratase (FH) gene germline mutation. It is speculated that for HRLCC infertility women with multiple uterine leiomyomas, preimplantation genetic testing may help block transmission of mutated FH gene during pregnancy.

**Case presentation:**

We present the case of a 26-year-old nulligravida with a history of early-onset uterine leiomyomatosis had a heterozygous nonsense mutation [NM_000143.4 (FH): c.1027C > T(p.Arg343Ter)] in the HRLLC gene. After ovulation induction and *in vitro* fertilization, preimplantation genetic testing for monogenic disorders (PGT-M) on embryos revealed the absence of the pathogenic allele in two blastomeres. Uterine fibroids were identified before embryo transfer, leading to a submucosal myomectomy and long period of pituitary suppression by Gonadotropin-releasing hormone analog (GnRHa). The patient achieved a healthy live birth after the second cycle of frozen–thawed embryo transfer.

**Conclusion:**

This case details the successful treatment of an infertile patient with an HRLLC family history, resulting in a healthy birth through myomectomy and PGT-M selected embryo transplantation. Our literature search indicates the first reported live birth after HRLLC-PGT-M.

## Introduction

Hereditary leiomyomatosis and renal cell cancer (HLRCC) is characterized by the development of multiple tumor types, including cutaneous leiomyoma, uterine leiomyoma, and papillary renal cell carcinoma type 2 (PRCC2). The onset period of HLRCC is usually from adolescence to adulthood, and the penetrance increases with age (1). Fumarate hydratase is encoded by the FH gene located on autochromosome 1q 42.3-43. In this study, an infertile patient caused by FH gene mutation with HLRCC family history was successfully born with a healthy infant who blocked the family inheritance of the FH gene mutation through PGT-M selective embryo transfer after repeated tumor removal surgery and GnRH-a downregulation. Live birth after HRLCC-PGT-M has not been reported internationally.

## Methods

**Figure 1:**
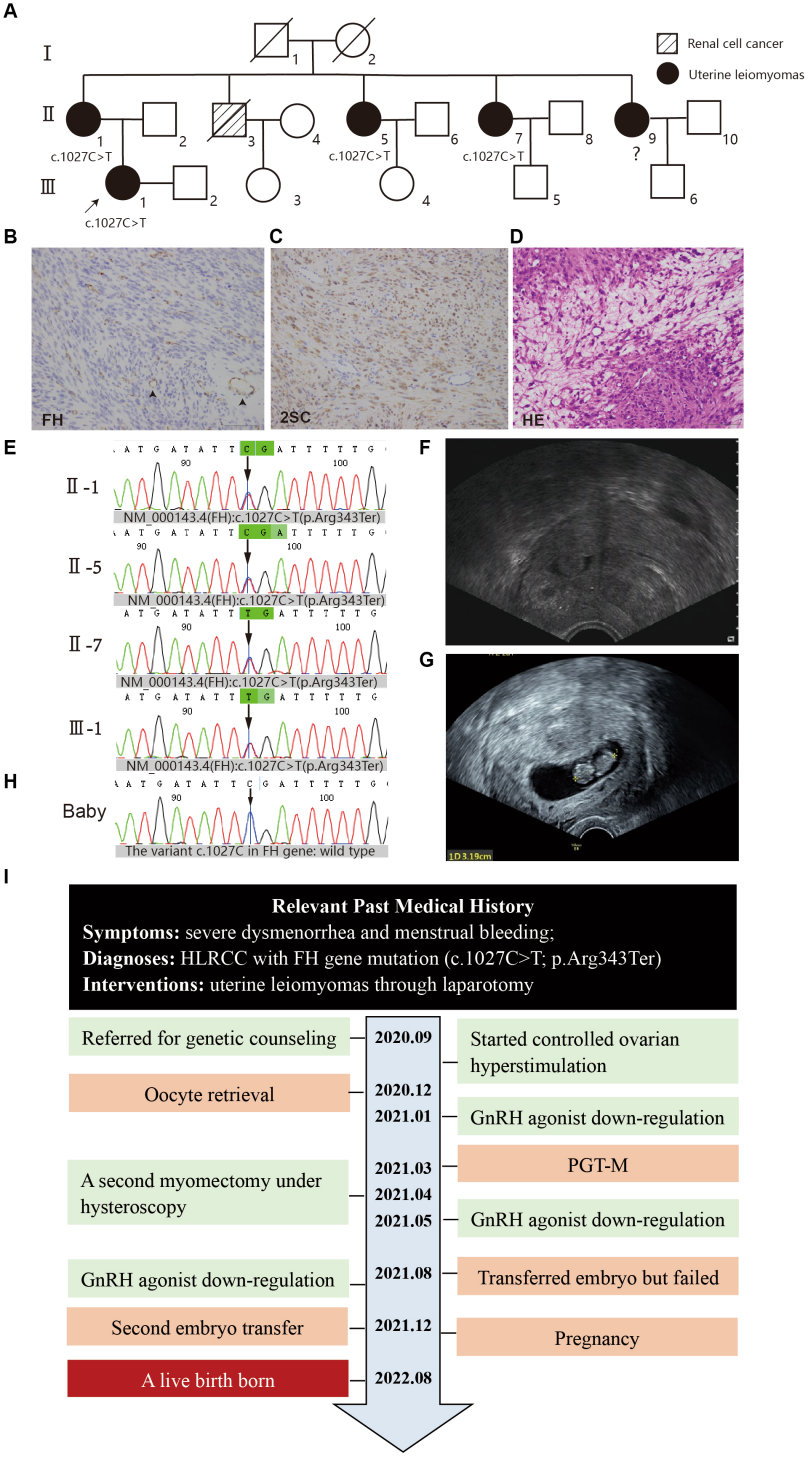
**(A)** Pedigree of the family. The white square/circle represents healthy male/female individuals; the oblique line means that individual has passed away. **(B–D)** Histopathology of uterine fibroids (×20), scale bars: 2 μm. **(B)** IHC for FH showed diffuse loss of FH staining in the cytoplasm of tumor cells in contrast with the presence of FH staining in blood vessel cells (arrowhead). **(C)** IHC for 2-succinocysteine (2SC) showed strong, diffuse cytoplasmic and nuclear staining. **(D)** Hematoxylin–eosin staining showed the cytoplasmic eosinophilic granules (arrow) and areas of patchy edema. **(E)** Mutation screening of the principle of family members using Sanger sequencing. The green bar indicates the mutation site (FH): c.1027C > T at an individual person. **(F)** The ultrasound showed in subsequent examination that myoma in the middle part of the anterior uterine wall compressed the uterine cavity. **(G)** The ultrasound showed a 5–6 weeks gestational sac. **(H)** Mutation screening of the baby through umbilical cord blood sampling. **(I)** The time line of this patient’s historical and current treatment.

This is a case report of a 26-year-old married female patient presented with early-onset multiple uterine fibroids due to severe dysmenorrhea and menstrual bleeding. In 2019, the patient underwent enucleation of multiple uterine leiomyomas through laparotomy and the intraoperative pathology assessment confirmed atypical leiomyoma. Immunohistochemical (IHC) staining of uterine fibroids showed 2SC positivity and FH loss (Figures 1B,C) and HE staining showed cell cytoplasm is eosinophilic (Figure 1D). Peripheral blood NGS confirmed heterozygous nonsense mutation of Exon 7 of the FH gene [NM_000143.4(FH): c.1027C > T(p.Arg343Ter)]. The patient had a family history in which her mother and three maternal aunts had all been diagnosed with uterine leiomyomas and treated with hysterectomy, while her maternal uncle had renal cell carcinoma and was deceased (Figure 1A). The patient’s family underwent whole exome sequencing and one mutation was identified [NM_000143.4(FH): c.1027C > T(p.Arg343Ter)], in accordance with recently updated guidelines (Figure 1E).

In November 2020, the patient, with a family history of HLRCC and FH gene mutation, was referred for genetic counseling. She inquired about *in vitro* fertilization with preimplantation genetic testing for monogenic diseases at our hospital. The patient had regular 28-day menstrual cycles, with normal hormone analysis. On cycle day 3, the baseline hormonal ovarian profile was as follows: AMH 9.8 ng/mL, FSH 8.0 IU/L, LH 3.8 IU/L, and testosterone 1.0 nmol/L. The partner was in good health and had a normal sperm analysis (concentration: 2.5 × 10^6^/mL, motility: 34.44% A + B). Both partners had normal karyotypes and tested negative for hepatitis B, hepatitis C, and HIV. However, transvaginal ultrasonography revealed numerous hypoechoic masses in the myometrium, predominantly in the anterior wall of the uterus, with the largest mass measuring 45 mm × 44 mm × 32 mm.

The patient was subjected to controlled ovarian hyperstimulation in an antagonist protocol, receiving 225 IU rFSH (Merck Serono) daily for 8 days. On the 5th day of stimulation, the GnRH antagonist Cetrolix (Merck Serono) was introduced at a dose of 0.25 mg daily for 3 days. On the 9th day, hCG 2,000 IU and Triptorelin 0.2 mg were administered to trigger follicular maturation. Ultrasound revealed a total of 14 visible follicles, with E2 levels at 13,411 pmol/L, LH levels at 0.2 IU/L, and P levels at 8.1 nmol/L.

Following hCG administration, oocyte retrieval was conducted using transvaginal ultrasound–guided aspiration, resulting in 13 oocytes with cumulus complexes. ICSI was performed on 12 oocytes for insemination, all of which fertilized successfully (see Table 1). Subsequently, PGT-M was applied to five embryos (four blastomeres and one blastocyst), revealing that three embryos carried pathological mutations while two were unaffected (see Table 2). After multidisciplinary consultation, a decision was made to transplant a single Day 3 blastomere.

**Table 1:** IVF cycle details.

IVF cycle details	First cycle
Oocytes retrieved	13
Oocyte fertilized	12
Blastocyst	1
Blastomere	4
Embryo biopsied	5
Heterozygous embryo	3
Homozygous embryo	2
Number of embryos transferred	2
Serum B-HCG	Positive

**Table 2:** PGT results.

Embryo ID	Biopsy cell type	Result	Details	Transferable
1	Blastomere	Heterozygous pathogenic	Inconclusive, −6, −9, −12, etc.^*^	No
6	Blastocyst	Heterozygous pathogenic	46 XN^**^	No
8	Blastomere	Heterozygous pathogenic	Inconclusive, −4, −5, −11, etc.^*^	No
10	Blastomere	Normal	46 XN^**^	Yes
12	Blastomere	Normal	46 XN^**^	Yes

PGT, Pre-implantation genetic testing; ^*^Chromosome abnormalities; ^**^N: X or Y chromosome.

In January 2021, the patient underwent a GnRH agonist downregulation protocol in preparation for embryo transfer. However, subsequent ultrasonography and abdominal MRIs revealed that a midsection fibroid in the anterior wall of the uterus was compressing the uterine cavity, impacting embryo implantation (Figure 1F). After discontinuing treatment, the patient underwent a second myomectomy under hysteroscopy in April. Following the surgery, she opted to proceed with embryo transfer. During a 3-month GnRH agonist downregulation treatment (with triptorelin acetate injected at a dose of 3.75 mg once every 4 weeks), one embryo was transferred. Unfortunately, she did not achieve pregnancy. After undergoing hormonally controlled cycles in preparation for another embryo transfer, the patient transferred a single embryo after 3 months. A positive β-hCG concentration of 1672.4 IU/L was detected, and subsequent ultrasound confirmation revealed a successful pregnancy (Figure 1G).

The patient declined any invasive prenatal diagnosis and opted for non-invasive prenatal testing (NIPT 2.0) at 15 weeks of gestation. The results indicated the absence of aneuploid abnormalities on Chromosomes 13, 18, and 21, and the sex chromosomes, as well as other chromosomes of the fetus. Additionally, no chromosome deletion/duplication syndrome was detected.

## Results

On August 27, 2022, the patient underwent a lower segment cesarean section (LSCS) and delivered a baby boy at 37 weeks and 2 days of gestation, weighing 3,240 g, with Apgar scores of 10. Umbilical cord blood sampling revealed that the neonate exhibited no chromosomal abnormalities and the variant c.1027C in FH gene was identified as wild type (Figure 1H). Meanwhile, she had a hysterectomy for placenta percreta, also to avoid hazards of rapidly growing fibroids. The timeline of the patient’s treatment is illustrated (Figure 1I). As of the last return visit, the child was 1 year old and demonstrated healthy development. The patient expressed high satisfaction with the treatment.

## Discussion

In this unique case, a patient with a classic FH gene mutation and early-onset multiple uterine fibroids underwent two myomectomies and embryo transfers, eventually giving birth to a healthy baby. This represents the first instance of a HRLCC patient successfully delivering a healthy baby. Despite searching multiple bibliographic databases and closely related articles, none of them reported live birth outcomes (2–4).

Preimplantation single gene genetic testing (PGT-M), coupled with assisted reproductive technology, effectively detects gene mutations, providing hope for healthy offspring in families at risk of hereditary diseases. However, the approach necessitates a collaborative effort among ART clinicians, embryologists, and molecular geneticists, demanding comprehensive counseling. High costs and ethical concerns pose barriers to widespread use. Long-term clinical practice is essential for postnatal verification. Our findings underscore the potential of PGT-M and may inspire protocols for various genetic disorders. Given the autosomal dominant nature of HRLCC, families seeking to minimize the risk of affected offspring should consider early genetic counseling and *in vitro* fertilization with PGT-M. Thorough patient consultation and risk communication are crucial in any PGT-M process. Obtaining informed consent before surgery and emphasizing clinical confirmation and prenatal genetic diagnosis are strongly recommended.

## Data availability statement

The datasets presented in this study can be found in online repositories. The names of the repository/repositories and accession number(s) can be found in the article/supplementary material.

## Ethics statement

The studies involving humans were approved by International Peace Maternity and Child Health Hospital Medical Ethics Committee. The studies were conducted in accordance with the local legislation and institutional requirements. Written informed consent for participation was not required from the participants or the participants’ legal guardians/next of kin in accordance with the national legislation and institutional requirements. Written informed consent was not obtained from the individual(s) for the publication of any potentially identifiable images or data included in this article because the participant has consented to the submission of the case report to the journal. Ethical approval was not required as this research letter are case reports.

## Author contributions

QH: Writing – review & editing, Writing – original draft, Visualization, Validation. QZ: Writing – review & editing, Methodology, Data curation. MG: Writing – original draft, Formal analysis, Data curation. HD: Writing – original draft, Methodology, Investigation. JX: Writing – review & editing, Resources, Methodology, Investigation. MZ: Writing – review & editing, Validation, Supervision, Conceptualization. MW: Writing – review & editing, Resources, Investigation, Data curation. LZ: Writing – review & editing, Methodology, Investigation. SL: Writing – review & editing, Resources, Investigation, Formal analysis. DW: Writing – original draft, Writing – review & editing, Visualization, Validation, Supervision, Funding acquisition, Conceptualization. WL: Writing – original draft, Writing – review & editing, Validation, Supervision.
